# Predicting major adverse cardiovascular events in diabetic and non-diabetic patients with coronary artery disease: visual models integrating multi-parametric coronary computed tomography angiography and pericoronary adipose tissue radiomics

**DOI:** 10.3389/fcvm.2026.1669037

**Published:** 2026-02-27

**Authors:** Ming Chen, Xiyi Huang, Lizhu Ouyang, Xinjie Chen, Jialing Pan, Liwen Wang, Lanni Zhou, Fusheng Ouyang, Qiugen Hu, Baoliang Guo

**Affiliations:** 1 Department of Radiology, The Eighth Affiliated Hospital of Southern Medical University, Foshan, Guangdong, China; 2 Department of Clinical Laboratory, Lecong Hospital of Shunde, Foshan, Guangdong, China

**Keywords:** coronary arteriosclerosis, coronary CT angiography, diabetes, major adverse cardiovascular events, pericoronary adipose tissue

## Abstract

**Objective:**

To compare the application value differences of PCAT radiomic features, clinical risk features and computed tomography (CT)-derived parameters in predicting Major adverse cardiovascular events (MACE) in patients with/without diabetes.

**Methods:**

Retrospective analysis included 1,000 coronary atherosclerosis patients undergoing Coronary CT angiography (CCTA) (with/without diabetes: 274/726) from the Eighth Affiliated Hospital of Southern Medical University. Clinical/CT data were collected, extracting 285 PCAT radiomic features from three major coronaries. Least absolute shrinkage and selection operator regression identified MACE-associated radiomic features. Patients underwent random 6:4 training/testing cohort split. Four predictive models were constructed: Model 1 (clinical factors), Model 2 (imaging factors), Model 3 (imaging-radiomic features), Model 4 (all factors).

**Results:**

In the training set, Model 4 showed the best performance: The area under the curves (AUC) of 0.803 [95% confidence interval (CI): 0.756–0.850] and 0.854 (95% CI: 0.779–0.929) for groups with/without diabetes, respectively. Model 3 outperformed Model 2 in patients without diabetes (*p* < 0.05), but not significantly in diabetic patients (*p* > 0.05).

**Conclusion:**

PCAT radiomics, CT-derived parameters, and plaque features demonstrate differential predictive value for MACE in patients with/without diabetes. Combining these with clinical risk factors provides most effective model for both.

## 1 Introduction

Diabetes mellitus (DM), a prevalent chronic metabolic disorder, demonstrates strong cardiovascular disease associations (1). Compared to non-diabetic individuals, the hyperglycemic state in DM promotes vascular calcification, exacerbates oxidative stress, and triggers inflammatory cytokine cascades, leading to more pronounced microcirculatory dysfunction and endothelial damage—thereby substantially increasing major adverse cardiovascular event (MACE) risks (1). These pathophysiological divergences result in distinct coronary artery disease progression patterns between diabetic and non-diabetic populations, necessitating comparative evaluations of cardiovascular risk assessment efficacy to inform clinical decision-making and prognosis improvement.

Coronary computed tomography angiography (CCTA) visualizes coronary anatomy but has limitations in functional assessment (2). CT-derived fractional flow reserve (CTFFR) improves stenosis diagnosis by integrating functional and anatomical data (2), while coronary artery calcium score (CACS) assesses plaque burden and MACE risk (3). However, in diabetic patients, the presence of microangiopathy and diffuse coronary calcification may lead to discrepancies between plaque burden and actual ischemic risk assessed by CTFFR and CACS, compared to non-diabetic individuals (4).

High-risk plaque (HRP) features, including low attenuation plaque (LAP), positive remodeling (PR), punctate calcification (PC) and napkin-ring sign (NRS), are strong predictors of MACE in coronary artery disease. They indicate plaque vulnerability, increasing risks of plaque rupture, thrombosis and rapid stenosis progression, thus elevating incidence of acute myocardial infarction, unstable angina and sudden cardiac death. Early identification via CCTA guides targeted therapy to reduce MACE and improve prognosis.

Emerging evidence indicates that pericoronary adipose tissue (PCAT) and vascular wall inflammation can exert reciprocal effects on one another, aiding in the identification of HRP progression (5–7). Fat Attenuation Index, derived from computed tomography (CT), quantifies PCAT density variations, providing insights into inflammation-related tissue alterations (8, 9). Elevated pericoronary adipose tissue attenuation (PCATA) is identified as a cardiac mortality risk factor (9, 10). However, changes in PCAT composition were associated not only with vascular inflammation but also with adverse fibrosis and remodeling of perivascular adipose tissue (7). Relying solely on CT attenuation may be insufficient to characterize PCAT complexity. Previous studies have suggested that higher levels of left anterior descending pericoronary adipose tissue attenuation (LAD-PCATA) may enhance the prediction of future adverse cardiovascular events in patients with DM (11).

PCAT are not confined to manifestations of vascular inflammation but are also closely linked to processes such as fibrosis and remodeling of the adipose tissue surrounding the coronary artery wall (7). Patients with DM often exhibit localized fibrosis and tissue remodeling in the perivascular adipose tissue, which is reflected as an increase in PCATA. Therefore, relying solely on CT attenuation values to evaluate PCAT alterations may fail to comprehensively capture its complex spatial architecture and physiological functions. Given this limitation, PCAT radiomics is an advanced field within cardiac radiology that leverages high-throughput, automated feature extraction to quantify the texture and composition of the fat surrounding coronary arteries on CCTA images, thereby enabling the analysis of high-dimensional imaging features for advanced assessment of coronary artery disease (CAD) (12).

Current methods for predicting MACE include PCAT radiomics, clinical factors, and CT-derived plaque characteristics and parameters. However, integrative analyses that combine these multimodal features remain relatively limited, and related visualization approaches are not yet fully explored. SHAP (SHapley Additive exPlanations), an interpretable machine learning tool, is still under investigation in this context and holds potential to enhance model interpretability.

This study aims to evaluate and compare the predictive value of PCAT radiomics features, clinical parameters, CT-derived metrics, and plaque characteristics for long-term MACE in patients with and without diabetes. By leveraging SHAP for visual interpretation, we seek to develop a refined risk stratification model to support clinical decision-making.

## 2 Material and methods

### 2.1 Patients

**Figure 1:**
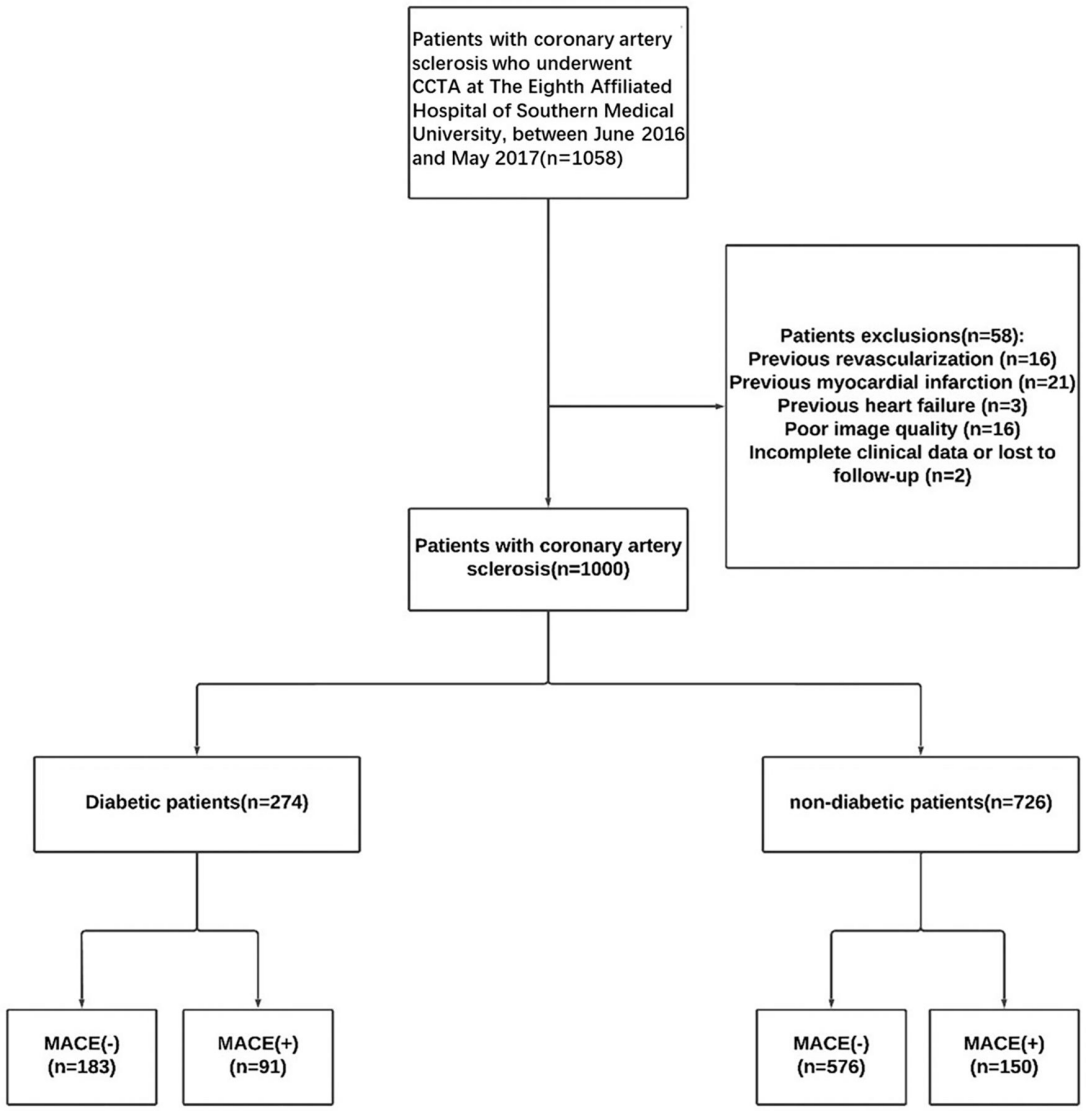
Flowchart showing inclusion and exclusion criteria for the study population. CCTA, coronary computed tomography angiography.

This retrospective study enrolled 1,000 patients with CAD (274 diabetic patients, 726 non- diabetic patients) from 1,058 initially screened at the Eighth Affiliated Hospital of Southern Medical University (June 2016-May 2017). The inclusion criteria were as follows: (1) Age ≥18 years; (2) CCTA-confirmed coronary artery disease with calcification; (3) Adequate image quality; (4) Measurable calcified plaques in major coronary arteries; (5) Complete baseline data; (6) Complete follow-up data. Exclusions comprised prior revascularization (*n* = 16), myocardial infarction (*n* = 21), heart failure (*n* = 3), poor image quality (*n* = 16), and incomplete data (*n* = 2). Patient selection process is detailed in Figure 1.

### 2.2 Assessment of clinical factors

Baseline characteristics, including prior medication history, demographic information (e.g., age, sex), and laboratory parameters such as hemoglobin A1c (HbA1c), total cholesterol (TC), low density lipoprotein cholesterol (LDL-C), high density lipoprotein cholesterol (HDL-C), non-high-density-lipoprotein cholesterol (non-HDL-C), residual cholesterol (RC), apolipoprotein A (Apo A), apolipoprotein B (Apo B), and monocyte count (Mono), were collaboratively extracted from clinical inpatient records by two radiologists. Hypertension was defined in accordance with established guidelines as a systolic blood pressure >140 mmHg and/or a diastolic blood pressure >90 mmHg, or by the current use of antihypertensive medications. DM was diagnosed based on the use of oral hypoglycemic agents or insulin, or a fasting blood glucose level ≥7.0 mmol/L. Smoking status was categorized as either current smoker or non-smoker.

### 2.3 Acquisition and analyses of CCTA

CCTA acquisition protocol is detailed in Supplementary Material.

The following parameters and plaque characteristics were manually assessed for major epicardial vessels with a diameter ≥ 2 mm: (1) affected coronary artery branches; (2) HRP characteristics, as defined by the following criteria: 1) PR, defined as any lesion with a remodeling index (the ratio of maximum lesion diameter to average diameter of normal proximal and distal vessel segments) ≥ 1.1; 2) LAP, defined as any region within a coronary plaque exhibiting attenuation < 30 Hu; 3) PC, characterized by calcific foci within the plaque with a diameter < 3 mm and a lesion perimeter < 90°; 4) the “ NRS”, defined as a plaque core with low CT attenuation surrounded by a dense peripheral zone.

Quantitative analysis of coronary plaques in epicardial vessels (≥2 mm) was performed using semi-automated software CoronaryDoc® (v1.11.1, Shukun, Beijing), which assessed volumes of lipid-rich, fibrofatty, fibrous, and calcified components, as well as diameter stenosis (DS), defined as (reference diameter−minimum lumen diameter)/reference diameter. CoronaryDoc® was also used to calculate CACS based on the Agatston method and estimate CTFFR using down-sampling and machine learning-based calibration techniques.

The concordance rate of the adverse plaque characteristics between two radiologists was determined using Cohen's kappa coefficient.

The concordance rate of DS between one radiologist and automated software was assessed using Intraclass Correlation Coefficient (ICC) and its 95% confidence interval was calculated in the same manner as that in the primary analysis.

### 2.4 Analysis of PCAT radiomic features

PCAT is defined as adipose tissue within a radial distance from the outer vascular wall equal to the coronary artery diameter, with CT attenuation values ranging from −190 to −30 Hu. PCAT radiomic phenotyping was performed on three coronary arteries per patient using the semi-automated software CoronaryDoc®. The surrounding adipose tissue around the proximal 40 mm segments of the left anterior descending and left circumflex arteries, and the 10–50 mm segment of the right coronary artery, was delineated. 95 radiomic features were extracted from each PCAT region, totaling 285 features, among which the PCAT attenuation index was included.

### 2.5 Follow-up and outcome

Follow-up information was retrieved via medical record review. Patients underwent at least one follow-up, with individual follow-up duration spanning from the CCTA date to the date of first MACE occurrence or the final data extraction date (May 2024). The median follow-up duration was 67.55 months [interquartile range (IQR), 30.02–86.01 months]. MACE was defined as a composite endpoint comprising cardiac death, congestive heart failure, re-hospitalization for unstable angina, non-fatal myocardial infarction, or coronary revascularization.

### 2.6 Model building and clinical application

Data for DM and non-DM groups was separately split into training and testing sets (6:4 ratio). The least absolute shrinkage and selection operator (LASSO) regression, with ten-fold cross-validation for optimal *λ*, selected key radiomic features. Non-zero coefficient features were then linearly combined, weighted by coefficients, to compute a radiomic score (Radscore) per patient.

Univariate Cox regression evaluated CT parameters and clinical features for predicting MACE. Variables with *p* < 0.1 entered multivariable Cox stepwise regression, with *p* < 0.05 considered statistically significant.

Extreme gradient boosting (XGBoost) models were consecutively established using only clinical factors (Model 1), only imaging indicators (Model 2), imaging indicators plus Radscore (Model 3), and all factors (Model 4) to predict MACE in both diabetic and non-diabetic patients. Models' performance was evaluated via the area under the curves (AUCs), compared using the DeLong test, and further assessed with time-dependent AUCs. SHapley Additive exPlanations (SHAP) values provide consistent, locally accurate attributions for each feature in each predictive model, enabling clearer interpretation of the machine learning model outcomes.

### 2.7 Statistical analysis

Continuous data normality was assessed using the Shapiro–Wilk test. Normally distributed variables were presented as mean ± SD, while non-normal data were reported as median (IQR). Comparisons were made using the t-test or Mann–Whitney U test. Categorical variables were expressed as frequencies and percentages, analyzed via the *χ*^2^ or Fisher's exact test. A multivariable Cox stepwise regression identified significant factors in diabetic and non-diabetic patients. ROC curves evaluated four models for predicting future MACE. Statistical analyses were performed using R software (version 4.4.1), with *p* < 0.05 considered significant.

## 3 Results

### 3.1 Comparison of MACE and non-MACE groups in diabetic and non-diabetic populations

This study included 1,000 coronary atherosclerosis patients (mean age 65.04 ± 10.46 years; 45.1% female), with 274 diagnosed with DM.

Interobserver agreement for assessing adverse plaque features was excellent, with Cohen's kappa (*κ*) values of 0.807 for PR, 0.861 for PC, 0.822 for LAP, and 0.807 for NRS. The manual and AI assessments of DS showed good agreement (ICC = 0.77; 95% CI, 0.72–0.81) (Supplementary Figure B1).

In the diabetic MACE group, patients exhibited more severe coronary stenosis, larger calcified plaque volumes, higher AST and creatinine levels, and a greater proportion of CTFFR ≤ 0.8 (Supplementary Tables C1–S2). In contrast, non-diabetic MACE patients showed older age, increased fibrous and calcified plaques, more severe stenosis, elevated ALT, lymphocytes, cholesterol, and triglycerides, along with higher rates of hypertension, medication use, NRS, CACS ≥ 100, and CTFFR ≤ 0.8, all with significant differences (Supplementary Tables C3–S4).

### 3.2 Models establishment

**Figure 2:**
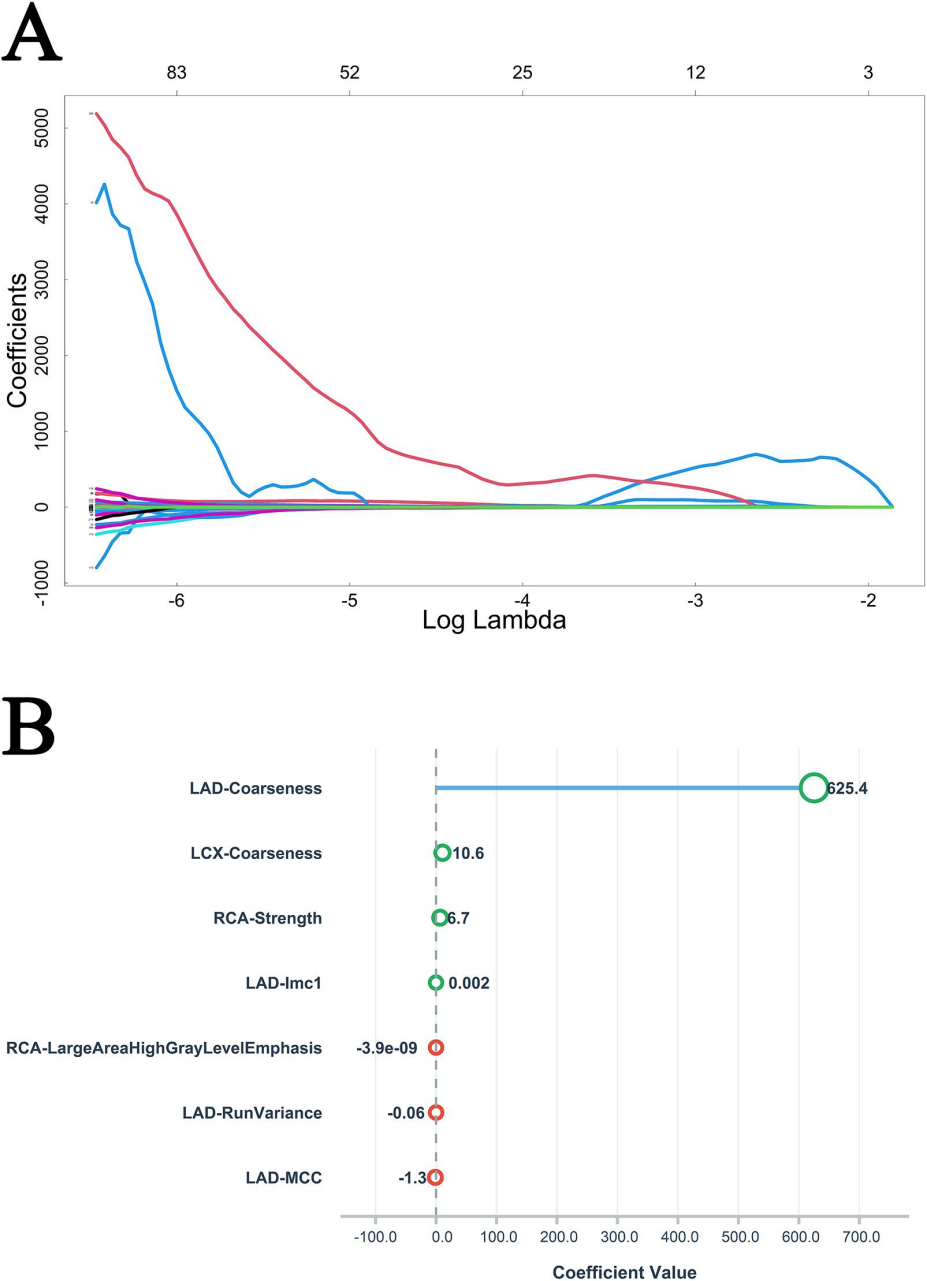
Variable selection path **(A)** and variable importance ranking **(B)** of LASSO regression analysis in DM group.

In the DM group, LASSO regression performed for feature selection (Figure 2A) identified three radiological features as significant characteristics (Figure 2B). Following univariate Cox screening (Supplementary Table C5), the multiple stepwise regression analysis selected five clinical factors and four imaging indicators as important risk factors (Table 1). Additionally, multivariable logistic regression indicated that ALP, creatinine, LDL, and CTFFR ≤ 0.8 were independent predictors of MACE in diabetic patients (all *p* < 0.05) (Table 1).

**Table 1:** Multivariate stepwise regression analysis in DM group.

Items	Hazard ratio (HR)
Female	0.63 (0.33–1.18, *p* = 0.15)
AST	1.01 (1.00–1.03, *p* = 0.01)
ALP	1.01 (1.00–1.01, *p* = 0.05)
Cr	1.00 (1.00–1.00, *p* < 0.01)
MONO	2.26 (0.91–5.64, *p* = 0.08)
CTFFR≤0.8	2.02 (1.06–3.85, *p* = 0.03)
PR	0.22 (0.07–0.74, *p* = 0.01)
Vlip	1.04 (1.00–1.08, *p* = 0.04)
Vcal	1.01 (1.00–1.01, *p* < 0.01)

*n*, number; CTFFR, computed tomography-derived fractional flow reserve; PR, positive remodeling; AST, aspartate aminotransferase; ALP, alkaline phosphatase; Cr, creatinine; MONO, monocytes; Vlip, lipid plaque volume; Vcal, calcified plaque volume; HRs with 95% CI and *p*.

**Figure 3:**
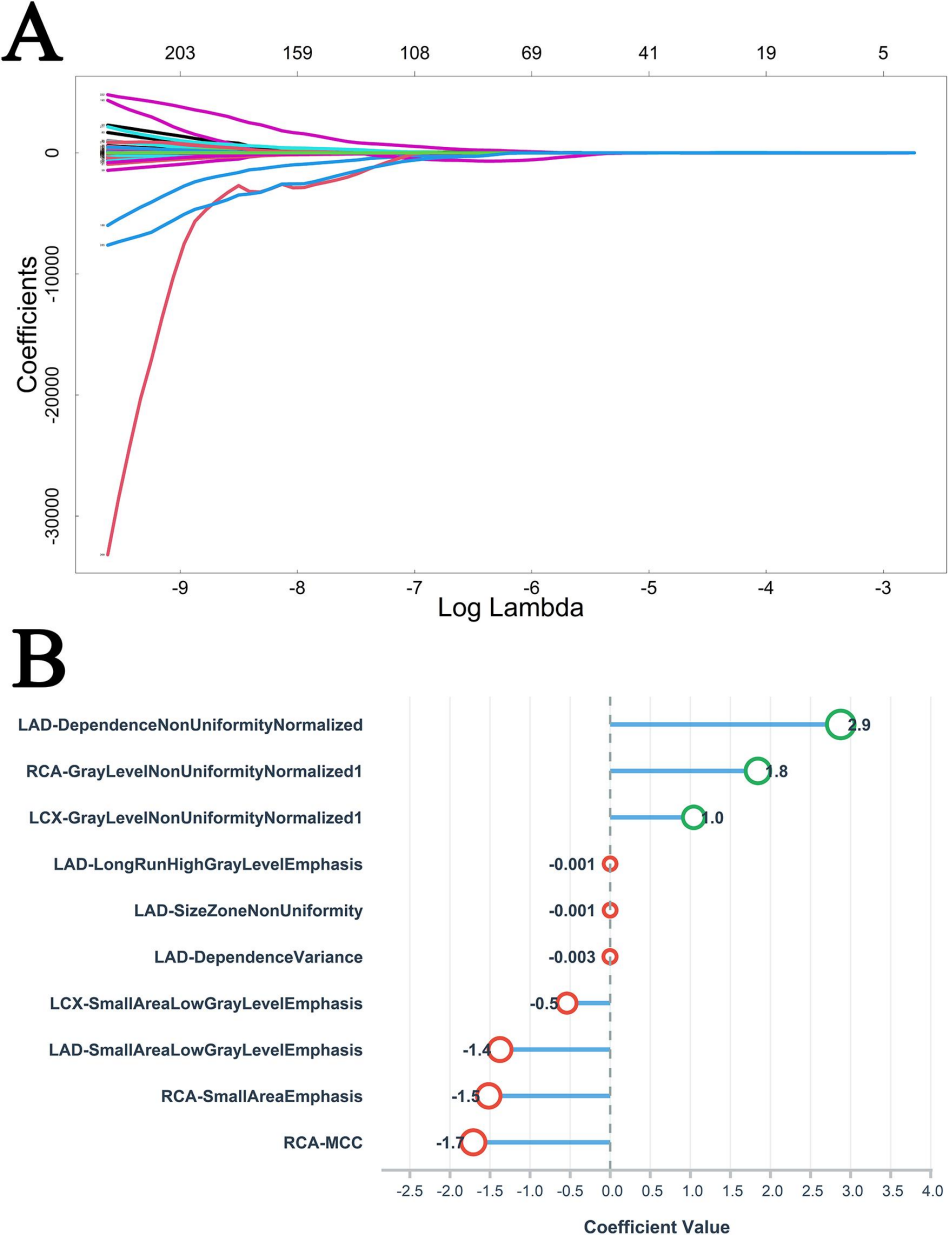
Variable selection path **(A)** and variable importance ranking **(B)** of LASSO regression analysis in non-DM group.

In the non-DM group, LASSO regression performed for feature selection (Figure 3A) identified 10 radiological features as significant characteristics (Figure 3B). Following univariate Cox screening (Supplementary Table C3), the multiple stepwise regression analysis revealed four clinical and five imaging risk factors, with age, TG, the volume of calcified plaque, CTFFR ≤ 0.8, and NRS as independent MACE predictors (*p* < 0.05) (Table 2).

**Table 2:** Multivariate stepwise regression analysis in non-DM groups.

Items	Hazard ratio (HR)
Age	1.03 (1.00–1.05, *p* = 0.02)
antihypertensive drugs	1.51 (0.94–2.44, *p* = 0.09)
Cr	1.00 (1.00–1.01, *p* = 0.09)
TG	0.73 (0.53–0.99, *p* = 0.04)
CTFFR≤0.8	1.80 (1.12–2.91, *p* = 0.02)
CACS≥100	1.48 (0.91–2.41, *p* = 0.12)
NRS	2.86 (1.25–6.56, *p* = 0.01)
legth	0.99 (0.97–1.01, *p* = 0.18)
Vcal	1.00 (1.00–1.01, *p* = 0.05)

*n*, number; CTFFR, computed tomography-derived fractional flow reserve; CACS, coronary artery calcium score; NRS, napkin-ring sign; Cr, creatinine; TG, triglycerides; Vcal, calcified plaque volume; HRs with 95% CI and *p*.

### 3.3 Comparison of models performance and pairwise

**Figure 4:**
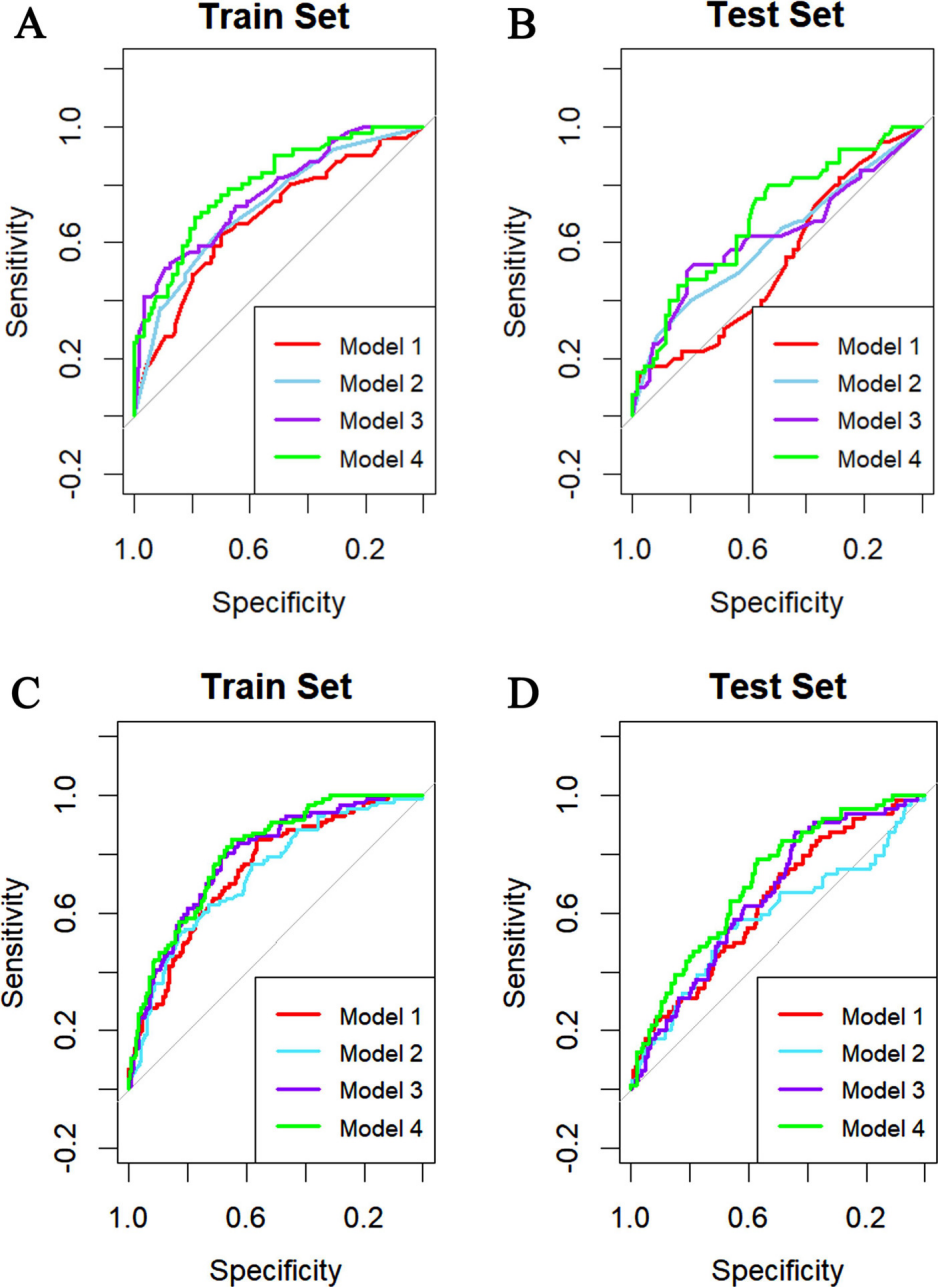
ROC curves for the four models in train set **(A)** and test set **(B)** of DM group and train set **(C)** and test set **(D)** of non-DM group.

In the DM group, ROC curves for the four models in both training and testing datasets are shown in Figures 4A, B. Model 4 outperformed Model 1, with Models 2 and 3 showing similar patterns. The AUCs for Model 4 in the training and testing datasets were 0.854 [95% confidence interval (CI): 0.779–0.929] and 0.706 (95% CI: 0.578–0.833), respectively, significantly higher than Model 1 (*p* < 0.05, DeLong test). These results suggest Model 4's potential as a quantitative tool for MACE prediction in DM patients.

In the non-DM group, ROC curves for the four models in both training and testing datasets are shown in Figures 4C, D. Model 4 showed the best performance, with AUCs of 0.803 (95% CI: 0.756–0.850) and 0.705 (95% CI: 0.6377–0.774), significantly higher than Models 1 and 2 (*p* < 0.05). Model 3 had AUCs of 0.788 (95% CI: 0.737–0.838) and 0.654 (95% CI: 0.582–0.725), significantly different from Model 2 (*p* < 0.05).

**Figure 5:**
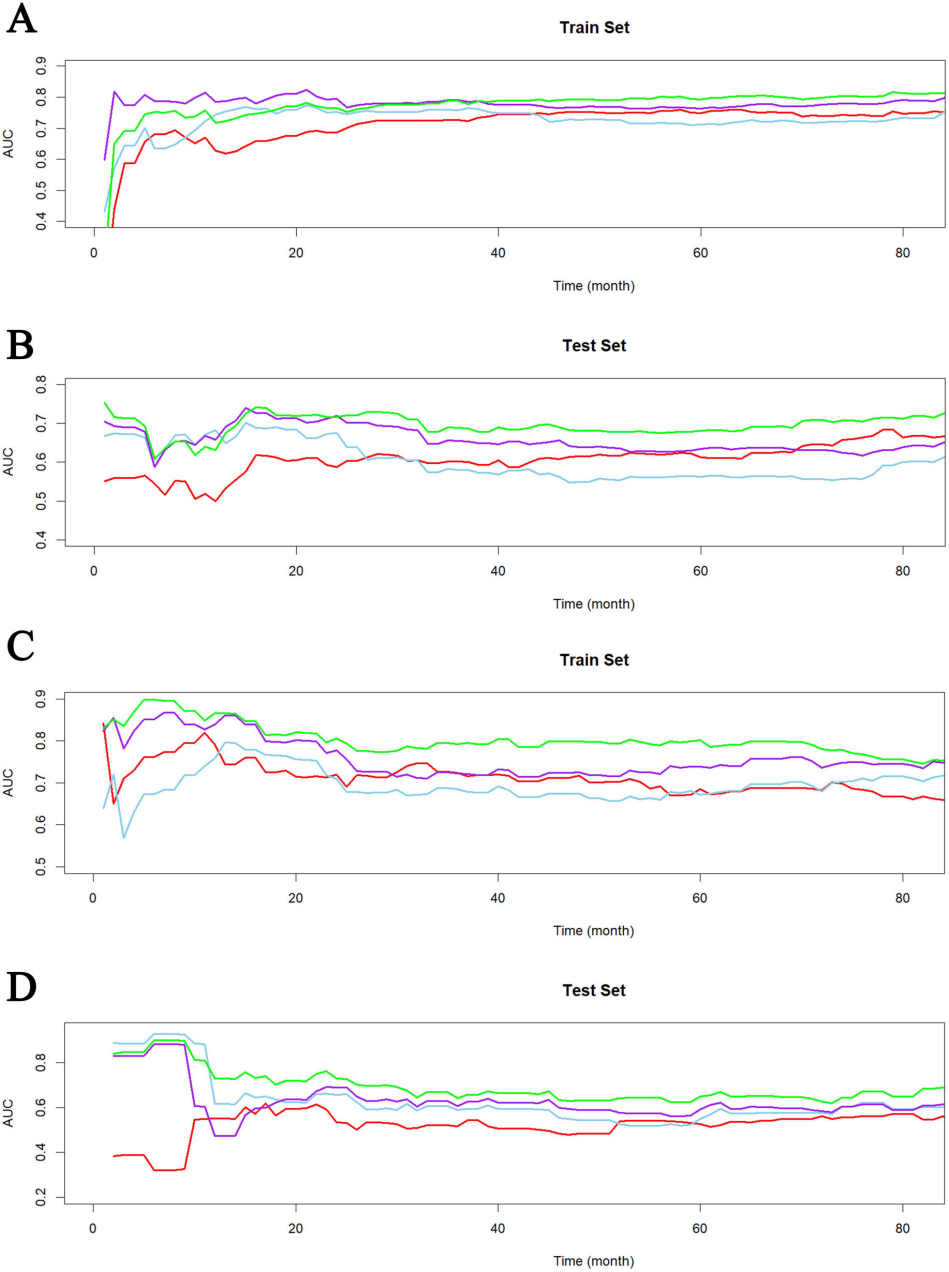
Time-dependent AUC curves in train set **(A)** and test set **(B)** of non-DM group and train set **(C)** and test set **(D)** of DM group.

As shown in Figure 5, Model 1 showed poor and unstable performance in both groups, while Model 4 demonstrated superior predictive accuracy across DM and non-DM populations.

### 3.4 Models explanation

In Model 4, SHAP values (Supplementary Figure C1) in diabetic patients identified calcified plaque volume as the strongest positive MACE predictor. PCAT radiomic score also ranked highly, followed by ALP, Cr and CTFFR≤0.8. Interactions emerged between CTFFR ≤ 0.8 and ALP, and low Cr synergized with CTFFR≤0.8. SHAP values rose with PCAT score around 0.5 and nonlinearly above 100 mm^3^ plaque (Supplementary Figure C2). CTFFR≤0.8 correlated with lipid plaque and PCAT scores, while female sex negatively correlated with PCAT scores (Supplementary Figure C4).

To evaluate the clinical utility of the predictive models, we performed decision curve analysis in the diabetic group at the 36-month time point (Supplementary Figure C6). The traditional clinical Model (Model 1) offered limited utility; In contrast, the combined model (Model 4) demonstrated superior clinical value. Although it exhibited some fluctuation in the 15%–20% range, it maintained a positive net benefit across a significantly wider range of threshold probabilities, extending up to approximately 45%.

As shown in Supplementary Figure C8A, both curves exhibited some fluctuations. However, the Model 4 generally demonstrated better agreement between predicted and observed probabilities compared to the Model 1, particularly in the high-risk spectrum. Although the Model 4 showed some deviation in the 10%–15% predicted probability range, it realigned well with the ideal diagonal line in the higher risk range, indicating its robustness in identifying high-risk diabetic patients. Model 1 exhibited poor generalizability in the validation cohort (Brier Score = 0.1815). However, the inclusion of conventional imaging indicators, and the PCAT radiomics score in Model 4 led to a observable reduction in prediction error (Brier Score = 0.1705).

In non-diabetics, Radscore, antihypertensive therapy, age, triglycerides and CTFFR≤0.8 were top contributors (Supplementary Figure C1). PCAT radiomic score and calcified plaque volume exhibited broad SHAP ranges with nonlinear, interactive effects. SHAP values increased stepwise with PCAT score and sharply with calcified plaque volume (Supplementary Figure C3). Low inter-variable correlations indicated predictor independence (Supplementary Figure C5).

We performed decision curve analysis to evaluate the clinical utility of the models specifically in the non-diabetic group at 36 months (Supplementary Figure C7). The traditional clinical model (Model 1) demonstrated suboptimal performance, while the combined model (Model 4) consistently provided the highest net benefit across a broad range of threshold probabilities (approx. 2% to 40%).

The calibration curves for 36-month survival in the non-diabetic group are shown in Supplementary Figure C8B. Due to the limited sample size in this subgroup, both curves exhibited moderate fluctuations when visualized using the nearest neighbor estimation method. While the Model 1 showed a lack of fit in the high-risk region, the Model 4 maintained an upward trajectory that paralleled the ideal diagonal more closely, indicating a superior capability to distinguish higher-risk individuals within this specific subgroup. Model 1 showed poor calibration and prediction accuracy in the validation cohort (Brier Score = 0.1275). In contrast, Model 4 significantly improved performance (Brier Score = 0.1172), demonstrating the added value of integrating conventional imaging indicators and the PCAT radiomics score into the clinical model.

## 4 Discussion

This study developed four models to predict MACE in coronary atherosclerosis patients with and without diabetes. Models combining clinical, PCAT radiomic, and CT features showed superior performance. In non-diabetic patients, adding radiomics improved prediction, while in diabetic patients, both PCAT radiomics and CT features were needed for enhancement. SHAP analysis interpreted the model 4, supporting clinical utility. Findings highlight the importance of personalized risk assessment for diabetic and non-diabetic populations.

### 4.1 Comparative application PCAT in predicting MACE in diabetic and non- diabetic patients

Our study showed that incorporating PCAT radiomic features improved diagnostic performance in the non-DM group, while in the DM group, only the combination of Radscore and CT imaging parameters enhanced accuracy. These findings highlight the differing roles of imaging biomarkers in CAD risk stratification between diabetic and non-diabetic populations.

In non- diabetic individuals, the added value of PCAT features likely reflects their ability to detect localized inflammation around coronary plaques, a key indicator of plaque vulnerability and vascular inflammation closely linked to MACE risk (13, 14). Our results support the predictive value of PCAT features in non- diabetic patients, who lack systemic inflammatory confounders, thus allowing localized markers to play a greater role.

In the DM group, only integrated models combining Radscore and CT imaging parameters yielded superior diagnostic performance. This highlights the more complex plaque composition and the influence of systemic inflammation in diabetes, aligning with earlier studies emphasizing the limitations of relying solely on PCAT features in this population (15). Multiparametric approaches have been recommended to improve prediction in diabetic patients, and our findings validate this strategy.

Although previous studies noted the utility of PCATA values in predicting MACE in diabetic patients (11, 16). Others reported that systemic inflammation may weaken the predictive value of PCAT features (15, 17). Our study applied more sensitive radiomic metrics and focused on the integrated effect of predictive models. While PCAT features alone had limited contribution, their combination with CT parameters significantly improved performance, offering new evidence in this context.

In short, PCAT radiomic features enhance risk prediction in non-diabetic patients by detecting localized inflammation, while diabetic patients benefit from models incorporating both focal and systemic imaging parameters.

### 4.2 Comparison of the predictive performance of CACS for MACE in diabetic and non- diabetic patients

This study found CACS ≥100 to be an independent predictor of MACE in non-DM patients, but not in diabetic patients, indicating a key difference in its predictive value. This may be due to differing coronary disease patterns: non- diabetic patients often exhibit calcified plaques, while diabetic patients show diffuse, non-calcified, and vulnerable plaques (4). Additionally, systemic inflammation, endothelial dysfunction, and metabolic abnormalities in DM may reduce the incremental value of CACS ≥100 (18, 19). Although some studies report improved risk prediction using CACS in diabetics over time (20), others highlight its limitations in certain diabetic subgroups (21). Aligning with our findings. These inconsistencies may stem from variations in populations and CACS thresholds. Our results suggest CACS ≥100 is effective for non-diabetic risk stratification, but has limited predictive value in diabetic patients, emphasizing the need for tailored strategies.

### 4.3 The predictive value of CTFFR for MACE in diabetic and non- diabetic patients

This study showed that CTFFR ≤ 0.8 predicts MACE in both diabetic and non-diabetic patients, highlighting its robust, diabetes-independent value. CTFFR assesses the functional significance of stenosis by integrating anatomical and hemodynamic data, enabling ischemia-related lesion detection. Our findings align with prior studies confirming CTFFR's predictive value in diverse populations (22) and its superiority over anatomical assessment for identifying functional ischemia (23, 24). Notably, CTFFR remains effective in diabetic patients despite microvascular complications, supporting its role as a reliable tool for risk stratification across populations.

### 4.4 The impact of different plaque component volumes on MACE varied between patients with and without DM

Our study demonstrated that in diabetic patients, both lipid and calcified plaque volumes are independent MACE predictors. Conversely, only calcified plaque volume predicted MACE in non-diabetic patients, highlighting distinct roles of plaque components in risk stratification. This aligns with the prior study showing greater lipid-rich plaque burden and progression in diabetic patients (25). For diabetic patients, lipid plaque volume's predictive value likely arises from diabetes-specific CAD pathophysiology: heightened inflammation, metabolic issues, and endothelial dysfunction contribute to lipid plaque vulnerability and MACE (26).

Calcified plaque volume in diabetic patients, while initially indicative of stabilization (27), ultimately reflects increasing plaque burden and stenosis, worsening prognosis. While CACS quantifies calcification, volumetric assessment offers more comprehensive plaque burden information, encompassing both lipid and calcified components, crucial for risk assessment in diabetic patients.

In non- diabetic patients, calcified plaque volume as the sole predictor suggests that the overall burden of calcified atherosclerosis is paramount in determining cardiovascular risk. Non- diabetic individuals may present with more focal disease where calcified plaques signal advanced, chronic ischemic burden, even with less prominent vulnerable lipid-rich plaques (28).

Our findings highlight pathophysiological differences in CAD between diabetic and non-diabetic patients, emphasizing the need for tailored risk assessment considering lipid and calcified plaque volumes.

### 4.5 The impact of different plaque characteristics on MACE varied between patients with and without DM

This study found that NRS independently predicts MACE in non-diabetic patients, while PR predicts MACE in diabetics with a paradoxical protective effect. These findings suggest divergent imaging markers for vulnerable plaques in different populations, reflecting differences in coronary pathophysiology.

In non-diabetics, NRS correlates with plaques prone to rupture. Conversely, the unexpected protective effect of PR in diabetic patients, despite its typical association with HRP in atherosclerosis (29), warrants consideration. This may suggest that in diabetes, PR could represent early vascular adaptation, reflecting compensatory dilation in early disease stages with lower overall risk, contrasting with the higher-risk negative/mixed remodeling observed in later stages (30).

The divergent roles of NRS and PR highlight distinct MACE drivers in diabetic vs. non-diabetic individuals, requiring tailored risk stratification and management.

In conclusion, our study shows that the predictive values of PCAT radiomic features, different CT-derived parameters for future MACE are different between diabetic and non-diabetic patients. A model based on PCAT radiomic features, clinical risk features, and CT-derived parameters has the highest efficacy in diagnosing the occurrence of MACE in patients with DM and without DM.

This study has several limitations, including the need for further validation through multi-center clinical trials, the use of MACE as a composite endpoint without detailed subgroup analyses, and potential biases due to its retrospective nature. Future research should focus on prospective studies and individual event analyses for improved accuracy.

## Data Availability

The raw data supporting the conclusions of this article will be made available by the authors, without undue reservation.
