# Supplementary material for: Predicting major adverse cardiovascular events in diabetic and non-diabetic patients with coronary artery disease: visual models integrating multi-parametric coronary computed tomography angiography and pericoronary adipose tissue radiomics
Source: Front Cardiovasc Med. 2026 Feb 27;13:1669037. doi: 10.3389/fcvm.2026.1669037 (PMC12982186; doi:10.3389/fcvm.2026.1669037)
Supplement: Supplementary file 1 [file Presentation1.pdf]

Supplementary material

Appendix A

1 Coronary CT Angiography (CCTA) Acquisition Protocol

1.1 Patient Preparation:

Patients with a heart rate >70 beats per minute (bpm) and no contraindications received oral beta-blockade (metoprolol 25-50 mg) prior to the scan. All patients were administered 0.5 mg of sublingual nitroglycerin approximately 5 minutes before image acquisition to promote coronary vasodilation. Patients were instructed and trained to perform a breath-hold to minimize motion artifacts during the scan.

1.2 Scanning Equipment:

All scans were performed using a second-generation 128-slice dual-source CT scanner (SOMATOM Definition Flash, Siemens Healthineers, Forchheim, Germany).

1.3 Coronary Artery Calcium Scoring (CACS):

A non-contrast, prospective ECG-triggered sequential acquisition was performed for calcium scoring prior to the angiographic phase. Scan parameters were as follows: tube voltage 120 kV, tube current 80 mA, slice thickness 3.0 mm. The scan range covered the entire heart from the carina to the diaphragm.

1.4 CCTA Acquisition:

Scan Range: From 1 cm below the tracheal bifurcation to the cardiac

apex.

Acquisition Mode: Based on heart rate and regularity, one of two prospectively ECG-triggered protocols was used: a high-pitch spiral acquisition ("Flash Spiral" mode, pitch = 3.2-3.4) for patients with a low and regular heart rate (<65 bpm), or a sequential axial acquisition for higher or irregular heart rates.

Technical Parameters: Automated tube voltage selection (CARE kV, reference 100 kV) and automated tube current modulation (CARE Dose4D, reference quality index of 20) were applied. The detector collimation was 2 × 64 × 0.6 mm with a gantry rotation time of 0.28 seconds.

Image Reconstruction: Raw data were reconstructed using a sinogram-affirmed iterative reconstruction algorithm (SAFIRE, strength level 3). Primary image sets were reconstructed with a slice thickness of 0.75 mm and an increment of 0.5 mm using a dedicated medium-sharp vascular convolution kernel (B26f). For sequential scans, the optimal reconstruction window (typically 60-75% of the R-R interval) was selected.

1.5 Contrast Agent Administration:

A dual-head power injector was used. A weight-adjusted bolus of 50-70 mL (approx. 0.7 mL/kg) of non-ionic iodinated contrast medium (Iopromide, 0.769 g/mL) was injected intravenously at a flow rate of 5.0 mL/s, followed by a 40 mL saline chaser at the same rate. Bolus tracking was performed with a region of interest in the ascending aorta, and the scan

was automatically initiated 6 seconds after the attenuation threshold reached 150 Hounsfield Units (HU).

1.6 Image Post-processing:

All images were transferred to a Picture Archiving and Communication System (PACS).

2 Anatomic Plaque Parameters Analysis

All coronary segments ≥ 2 mm were evaluated according to an 18-segment model. Each coronary lesion was manually assessed for adverse plaque characteristics: (1) affected coronary artery branches; (2) HRP characteristics, as defined by the following criteria: 1) positive remodeling(PR), defined as any lesion with a remodeling index ≥ 1.1; 2) low-attenuation plaque(LAP), defined as any region within a coronary plaque exhibiting attenuation < 30 Hu; 3) punctate calcification(PC), characterized by calcific foci within the plaque with a diameter < 3 mm and a lesion perimeter < 90°; 4) the "napkin ring sign (NRS)", defined as a plaque core with low CT attenuation surrounded by a dense peripheral zone.

Quantitative analysis of coronary plaques in epicardial vessels (≥2 mm) was performed using semi-automated software CoronaryDoc® (v1.11.1, Shukun, Beijing), which assessed volumes of lipid-rich, fibrofatty, fibrous, and calcified components, as well as diameter stenosis (DS), defined as (reference diameter − minimum lumen diameter)/reference diameter. CoronaryDoc® was also used to calculate CACS based on the

Agatston method and estimate CTFFR using down-sampling and machine learning-based calibration techniques.

3 Diagnostic Definitions

Hypertension and DM were defined according to established guidelines[1]. Smoking status was categorized as current smoker or non-smoker.

4 PCAT Radiomic Analysis

The PCAT radiomic analysis protocol involved standardized segmentation of pericoronary adipose tissue surrounding specific coronary artery segments. The left anterior descending and left circumflex arteries were analyzed along their proximal 40-mm segments, while the right coronary artery was evaluated from 10 to 50 mm distal to its origin. All segmentations were performed using the semi-automated CoronaryDoc® software, which enabled consistent delineation of the adipose tissue compartment while accounting for anatomical variations. The extracted radiomic features encompassed a wide spectrum of texture characteristics, intensity distributions, and morphological patterns that collectively provide a comprehensive phenotypic characterization of the PCAT microenvironment.

Appendix B

Consistency Analysis

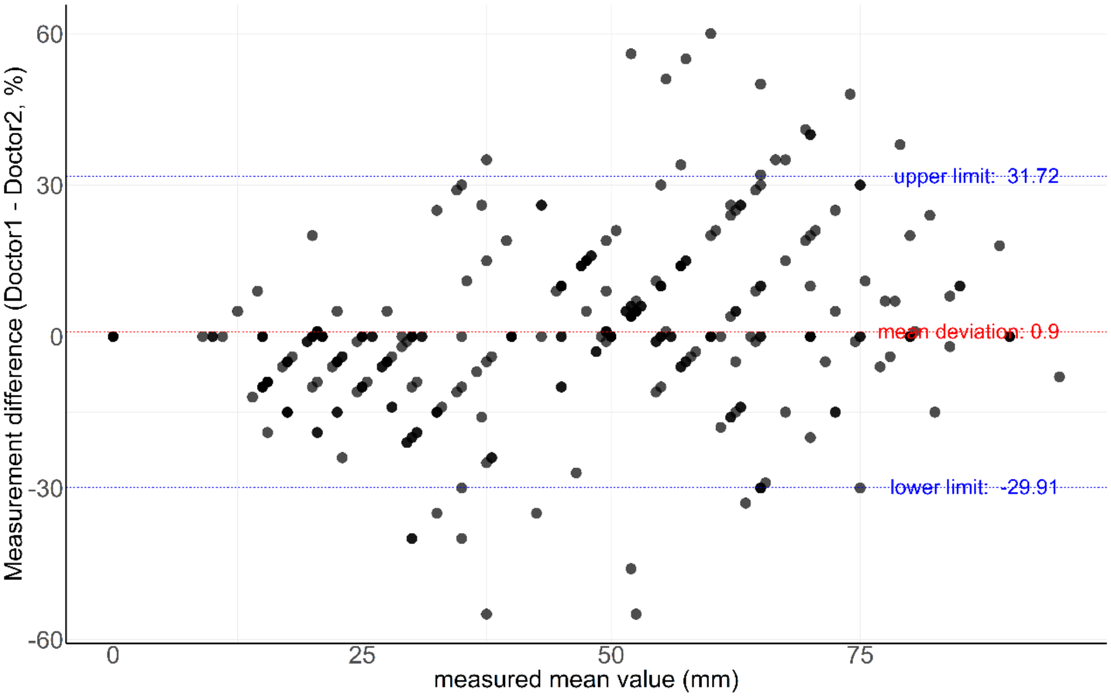

Supplemental Fig. B1 The Bland-Altman diagram of DS shows good consistency among observers.

Appendix C

Supplemental Table C1 Comparison of Baseline Characteristics Between the MACE and Non-MACE Groups in the DM Group.

	MACE(-) n=183	MACE(+) n=91	t/z/χ2	p
Female, n(%)	94.0(51.4)	35.0(38.5)	0.262	0.059
age(years)	65.85±9.66	68.13±9.41	0.24	0.064
HbA1c(%)	6.70(6.20,7.70)	6.70(6.25,7.85)	0.075	0.606
ALT(U/L)	21.00(16.00,32.00)	25.00(17.00,38.00)	0.232	0.153
AST(U/L)	21.00(16.00,27.00)	23.00(17.00,32.00)	0.298	0.021
GGT(U/L)	27.00(19.00,41.50)	30.00(20.00,55.50)	0.260	0.103
ALP(U/L)	75.00(59.50,91.50)	79.00(66.50,91.50)	0.260	0.105
Cr(μmol/L)	79.00(63.95,94.80)	89.60(69.00,109.55)	0.351	0.002
TC(mmol/L)	4.92(4.26,5.84)	4.80(3.88,5.72)	0.126	0.281
TG(mmol/L)	1.29(0.86,1.99)	1.37(1.03,2.15)	0.134	0.185

HDL-C(mmol/L)	1.57(1.21,2.66)	1.67(1.20,2.68)	0.011	0.889
LDL-C(mmol/L)	1.99(1.38,2.94)	1.68(1.22,2.61)	0.247	0.072
Non-HDL-C(mmol/L)	2.44(1.88, 3.44)	2.57(1.94, 3.65)	0.077	0.538
apoA(g/L)	1.37(1.16,1.62)	1.34(1.17,1.58)	0.082	0.507
apoB(g/L)	1.02±0.46	0.96±0.33	0.138	0.31
LYMPH(10^9/L)	1.88(1.44,2.42)	1.90(1.35,2.32)	0.142	0.663
MONO(10^9/L)	0.43(0.33,0.56)	0.43(0.32,0.58)	0.146	0.835
NEUT(10^9/L)	4.05(3.29,5.31)	4.28(3.20,5.40)	0.071	0.949
PLT(10^9/L)	216.00(183.00,250.00)	209.00(165.50,253.00)	0.038	0.477
hypertension(%)	160.0(87.4)	82.0(90.1)	0.085	0.652
antihypertensive drug(%)	124.0(67.8)	71.0(78.0)	0.232	0.104
antidiabetic drug(%)	119.0(65.0)	66.0(72.5)	0.162	0.266

Note: *n*, number; MACE, major adverse cardiovascular events; HbA1c, Hemoglobin A1c; ALT, Alanine Aminotransferase; AST, Aspartate Aminotransferase; GGT, Gamma-Glutamyl Transferase; ALP, Alkaline Phosphatase; Cr, Creatinine; TC, Total Cholesterol; TG, Triglycerides; HDL-C, High-Density Lipoprotein Cholesterol; LDL-C, Low-Density Lipoprotein Cholesterol; Non-HDL-C, Non-High-Density Lipoprotein Cholesterol ;apoA, Apolipoprotein A; apoB, Apolipoprotein B; LYMPH, Lymphocytes; MONO, Monocytes; NEUT, Neutrophils; PLT, Platelets. Data are presented as median (interquartile range) or number (percentage) as appropriate.

Supplemental Table C2 Comparison of Imaging Parameters Between the MACE and Non-MACE Groups in the DM Group.

	MACE(-) n=183	MACE(+) n=91	t/z/χ2	p
CTFFR≤0.8 (%)	55.0(30.1)	47.0(51.6)	0.45	0.001
CACS≥100(%)	117.0(63.9)	63.0(69.2)	0.112	0.463
PR(%)	33(18.0)	14(15.4)	0.071	0.706
PC(%)	62(33.9)	35(38.5)	0.095	0.540
LAP(%)	37.0(20.2)	20.0(22.0)	0.043	0.857
NRS(%)	10.0(5.5)	9.0(9.9)	0.167	0.269
length(mm)	16.80(8.09, 28.90)	24.61(12.84, 39.34)	0.356	0.002
DS(%)	45.00(21.00,65.00)	59.00(27.50,75.50)	0.483	<0.001
Vnon-cal(mm3)	30.15(12.85, 65.94)	40.24(19.27, 80.83)	0.240	0.059

Vlip(mm3)	2.26(0.14,8.36)	4.04(0.76,10.94)	0.187	0.068
Vfrlip(mm3)	12.21(4.07,32.56)	15.18(3.17,35.83)	0.196	0.292
Vfr(mm3)	13.68(5.46,25.44)	17.92(6.87,35.75)	0.247	0.086
Vcal(mm3)	7.78(0.60,26.83)	27.69(5.30,75.28)	0.462	<0.001

Note: *n*, number; CTFFR, computed tomography-derived fractional flow reserve; CACS, coronary artery calcium score; MACE, major adverse cardiovascular events; PR, positive remodeling; LAP, low-attenuation plaque; PC, punctate calcification; NRS, napkin ring sign; DS, Diameter stenosis; Vlip, Lipid Plaque Volume; Vfrlip, Fibrofatty Plaque Volume; Vfr, Fibrous Plaque Volume; Vcal, Calcified Plaque Volume. Data are presented as median (interquartile range) or number (percentage) as appropriate.

Supplemental Table C3 Comparison of Baseline Characteristics Between the MACE and Non-MACE Groups in the Non-DM Group.

	MACE(-) n=576	MACE(+) n=150	t/z/χ2	p
Female, n(%)	256.0(44.4)	66.0(44.0)	0.009	0.996
age(years)	63.21±10.55	69.20±10.00	0.583	<0.001
HbA1c(%)	5.80(5.50,6.10)	5.72(5.50,6.10)	0.038	0.24
ALT(U/L)	22.00(16.00,31.00)	19.00(15.00,29.75)	0.036	0.026
AST(U/L)	21.00(17.00,27.00)	21.00(18.00,26.00)	0.01	0.587
GGT(U/L)	26.00(18.00,41.25)	25.00(17.00,42.75)	0.065	0.481
ALP(U/L)	73.00(58.00,93.00)	72.00(57.25,93.50)	0.029	0.495
Cr(μmol/L)	81.55(66.95,96.48)	84.70(65.85,100.85)	0.234	0.223
TC(mmol/L)	5.16(4.42,5.99)	4.88(4.07,5.74)	0.004	0.012
TG(mmol/L)	1.28(0.88,1.76)	1.07(0.79,1.57)	0.275	0.004
HDL-C(mmol/L)	1.68(1.26,2.76)	1.56(1.25,2.46)	0.08	0.339
LDL-C(mmol/L)	2.13(1.42,3.28)	2.02(1.48,2.95)	0.132	0.373
Non-HDL-C(mmol/L)	2.88 (2.18, 4.07)	2.66 (2.12, 3.77)	0.117	0.003
apoA(g/L)	1.41(1.19,1.66)	1.35(1.14,1.63)	0.071	0.222
apoB(g/L)	1.09±1.17	0.94±0.30	0.151	0.14
LYMPH(10^9/L)	1.85(1.43,2.28)	1.60(1.23,2.18)	0.101	0.007
MONO(10^9/L)	0.43(0.31,0.56)	0.44(0.33,0.59)	0.031	0.563
NEUT(10^9/L)	3.53(2.88,4.60)	3.77(2.82,4.67)	0.08	0.662

PLT(10^9/L)	216.00(183.00,251.00)	203.50(171.25,249.25)	0.107	0.076
hypertension(%)	404.0(70.1)	118.0(78.7)	0.196	0.049
antihypertensive drugs (%)	269.0(46.7)	95.0(63.3)	0.339	<0.001

Note: *n*, number; MACE, major adverse cardiovascular events; PR, positive remodeling; LAP, low-attenuation plaque; PC, punctate calcification; NRS, napkin-ring sign; HbA1c, hemoglobin A1c; ALT, alanine aminotransferase; AST, aspartate aminotransferase; GGT, gamma-glutamyl transferase; ALP, alkaline phosphatase; Cr, creatinine; TC, total cholesterol; TG, triglycerides; HDL-C, high-density lipoprotein Cholesterol; LDL-C, low-density lipoprotein Cholesterol; Non-HDL-C, Non-High-Density Lipoprotein Cholesterol; apoA, apolipoprotein A; apoB, apolipoprotein B; LYMPH, lymphocytes; MONO, monocytes; NEUT, neutrophils; PLT, platelets. Data are presented as median (interquartile range) or number (percentage) as appropriate.

Supplemental Table C4 Comparison of Imaging Parameters Between the MACE and Non-MACE Groups in the Non-DM Group.

	MACE(-) n=576	MACE(+) n=150	t/z/χ2	p
CTFFR≤0.8 (%)	129.0(22.4)	60.0(40.0)	0.413	<0.001
CACS≥100(%)	339.0(58.9)	109.0(72.7)	0.294	0.003
PR(%)	68.0(11.8)	22.0(14.7)	0.085	0.419
PC(%)	180.0(31.2)	51.0(34.0)	0.059	0.585
LAP(%)	97.0(16.8)	31.0(20.7)	0.098	0.33
NRS(%)	12.0(2.1)	11.0(7.3)	0.25	0.003
length(mm)	14.02(7.04, 24.69)	19.35(10.20, 31.15)	0.257	0.001
DS(%)	27.50(20.00,57.00)	55.00(25.00,74.00)	0.412	<0.001
Vnon-cal(mm3)	27.38 (9.13, 58.03)	28.84 (11.99, 68.72)	0.108	0.153
Vlip(mm3)	1.71(0.00,6.35)	1.88(0.00,6.85)	0.025	0.813
Vfrlip(mm3)	9.84(1.68,25.42)	9.56(1.27,30.74)	0.018	0.789
Vfr(mm3)	12.05(4.02,23.76)	16.17(5.68,29.20)	0.251	0.014
Vcal(mm3)	7.23(0.06,25.80)	18.20(2.45,60.30)	0.391	<0.001

Note: *n*, number; CTFFR, computed tomography-derived fractional flow reserve; CACS, coronary artery calcium score; MACE, major adverse cardiovascular events;

PR, positive remodeling; LAP, low-attenuation plaque; PC, punctate calcification; NRS, napkin-ring sign; DS, diameter stenosis; Vlip, lipid plaque volume; Vfrlip, fibrofatty plaque volume; Vfr, fibrous plaque volume; Vcal, calcified plaque volume. Data are presented as median (interquartile range) or number (percentage) as appropriate.

Supplemental Table C5 Univariable regression analysis in the DM group.

	Hazard Ratio in DM group	Hazard Ratio in non-DM group
female	0.53 (0.29-0.95, p=0.03)	0.97 (0.63-1.48, p=0.88)
age	1.01 (0.98-1.04, p=0.57)	1.04 (1.02-1.06, p<0.01)
hypertension	1.89 (0.59-6.06, p=0.29)	1.76 (0.99-3.12, p=0.06)
antihypertensive drug	1.08 (0.57-2.07, p=0.81)	2.05 (1.30-3.24, p<0.01)
antidiabetic drug	1.15 (0.65-2.04, p=0.64)	-
HbA1c	1.02 (0.83-1.26, p=0.83)	0.83 (0.65-1.07, p=0.16)
ALT	1.01 (1.00-1.02, p=0.16)	0.99 (0.98-1.01, p=0.33)
AST	1.01 (1.00-1.02, p<0.01)	1.00 (0.99-1.01, p=0.56)
GGT	1.00 (1.00-1.00, p=0.07)	1.00 (0.99-1.00, p=0.91)
ALP	1.01 (1.00-1.01, p<0.01)	1.00 (0.99-1.01, p=0.95)
Cr	1.00 (1.00-1.00, p<0.01)	1.00 (1.00-1.01, p=0.01)
TC	0.98 (0.79-1.21, p=0.84)	1.00 (1.00-1.00, p=0.92)
TG	1.03 (0.86-1.23, p=0.78)	0.70 (0.52-0.95, p=0.02)
HDL-C	0.84 (0.63-1.11, p=0.22)	1.07 (0.87-1.31, p=0.53)
LDL-C	1.04 (0.82-1.31, p=0.77)	0.85 (0.70-1.04, p=0.11)
Non-HDL-C	0.95 (0.75-1.19, p=0.633)	1.00 (1.00-1.00, p=0.921)
apoA	0.63 (0.24-1.68, p=0.36)	1.27 (0.73-2.22, p=0.41)
apoB	0.90 (0.45-1.81, p=0.77)	0.82 (0.52-1.31, p=0.41)
LYMPH	0.90 (0.62-1.31, p=0.59)	0.75 (0.54-1.04, p=0.08)
MONO	2.62 (1.18-5.79, p=0.02)	0.90 (0.43-1.91, p=0.79)
NEUT	1.03 (0.91-1.17, p=0.62)	1.04 (0.94-1.15, p=0.50)
PLT	1.00 (1.00-1.00, p=0.60)	1.00 (0.99-1.00, p=0.27)
CTFFR≤0.8	2.62 (1.49-4.60, p<0.01)	2.06 (1.33-3.18, p<0.01)
CACS≥100	1.56 (0.82-2.98, p=0.18)	1.79 (1.12-2.87, p=0.02)
PR	0.41 (0.15-1.13, p=0.08)	1.17 (0.62-2.21, p=0.63)
PC	1.16 (0.66-2.04, p=0.60)	1.00 (0.63-1.58, p=1.00)
LAP	1.13 (0.59-2.16, p=0.71)	0.93 (0.54-1.59, p=0.78)
NRS	0.76 (0.24-2.43, p=0.64)	3.56 (1.64-7.72, p<0.01)
legth	1.01 (1.00-1.03, p=0.01)	1.01 (1.00-1.02, p=0.06)
DS	1.02 (1.01-1.03, p<0.01)	1.01 (1.00-1.01, p=0.12)
Vnon-cal	1.01 (1.00-1.01, p=0.024)	1.00 (1.00-1.01, p=0.845)

Vlip	1.04 (1.01-1.06, p<0.01)	0.99 (0.97-1.02, p=0.62)
Vfrlip	1.01 (1.00-1.02, p=0.08)	1.00 (0.99-1.01, p=0.62)
Vfr	1.01 (1.00-1.03, p=0.04)	1.01 (1.00-1.02, p=0.12)
Vcal	1.01 (1.00-1.01, p<0.01)	1.00 (1.00-1.01, p<0.01)

Note: *n*, number; CTFFR, computed tomography-derived fractional flow reserve; CACS, coronary artery calcium score; LAP, low-attenuation plaque; PC, punctate calcification; NRS, napkin-ring sign; DS, diameter stenosis; HbA1c, hemoglobin A1c; ALT, alanine aminotransferase; AST, aspartate aminotransferase; GGT, gamma-glutamyl transferase; ALP, alkaline phosphatase; Cr, creatinine; TC, total cholesterol; TG, triglycerides; HDL-C, High-Density Lipoprotein Cholesterol; LDL-C, low-density lipoprotein Cholesterol; Non-HDL-C, Non-High-Density Lipoprotein Cholesterol; apoA, apolipoprotein A; apoB, apolipoprotein B; LYMPH, lymphocytes; MONO, monocytes; NEUT, neutrophils; PLT, platelets; SD, standard deviation; Vlip, lipid plaque volume; Vfrlip, fibrofatty plaque volume; Vfr, fibrous plaque volume; Vcal, calcified plaque volume. Hazard ratios are presented with 95% confidence intervals and corresponding *p* values in parentheses.

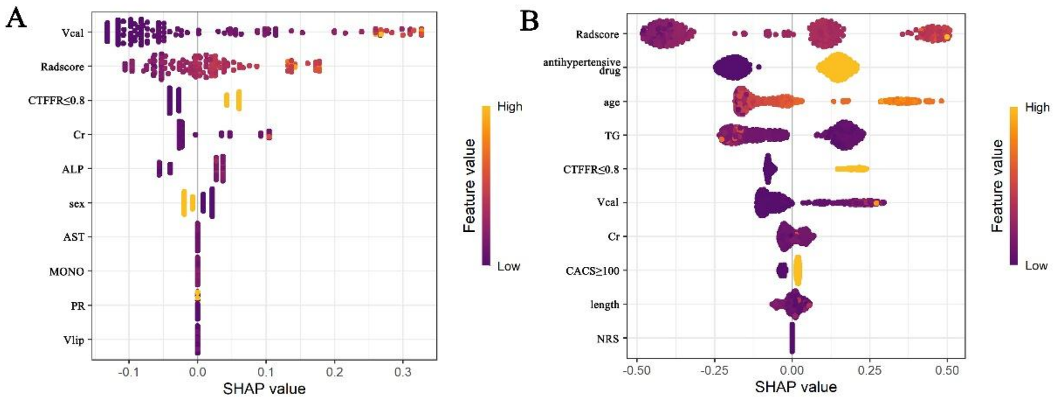

Supplemental Fig. C1 The top 10 risk factors for major adverse cardiovascular events in the diabetic (A) and non-diabetic (B) groups were ranked based on their importance, as determined by the optimal predictive model, with red dots for high risk and blue dots for low risk. SHAP, SHapley Additive exPlanations; Radscore, radiomic score; CTFFR, computed tomography-derived fractional flow reserve; AST, aspartate aminotransferase; ALP, alkaline phosphatase; Cr, creatinine; Vcal, calcified plaque volume; Vlip, lipid plaque volume.

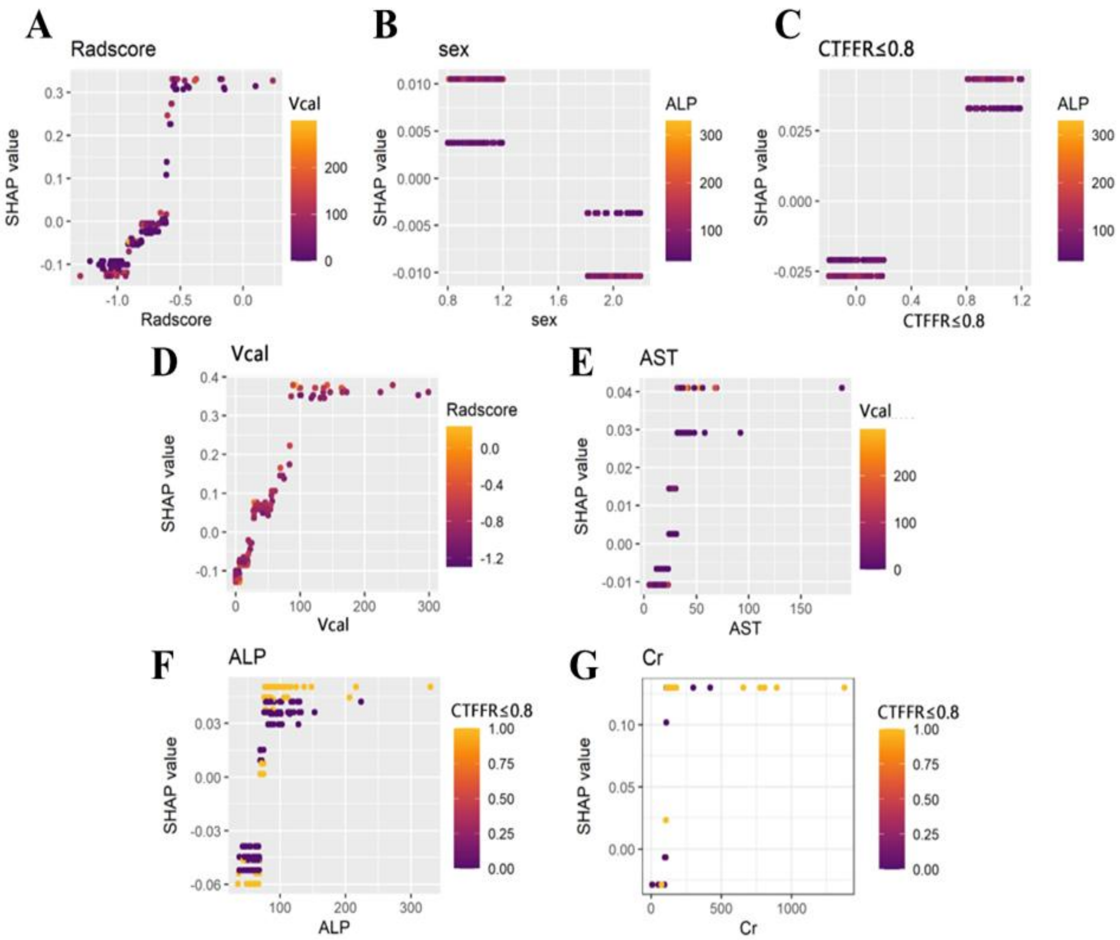

Supplemental Fig. C2 SHAP dependence plots in the diabetic group. SHAP, SHapley Additive exPlanations; Radscore, radiomic score; CTFFR, computed tomography-derived fractional flow reserve; AST, aspartate aminotransferase; ALP, alkaline phosphatase; Cr, creatinine; Vcal, calcified plaque volume.

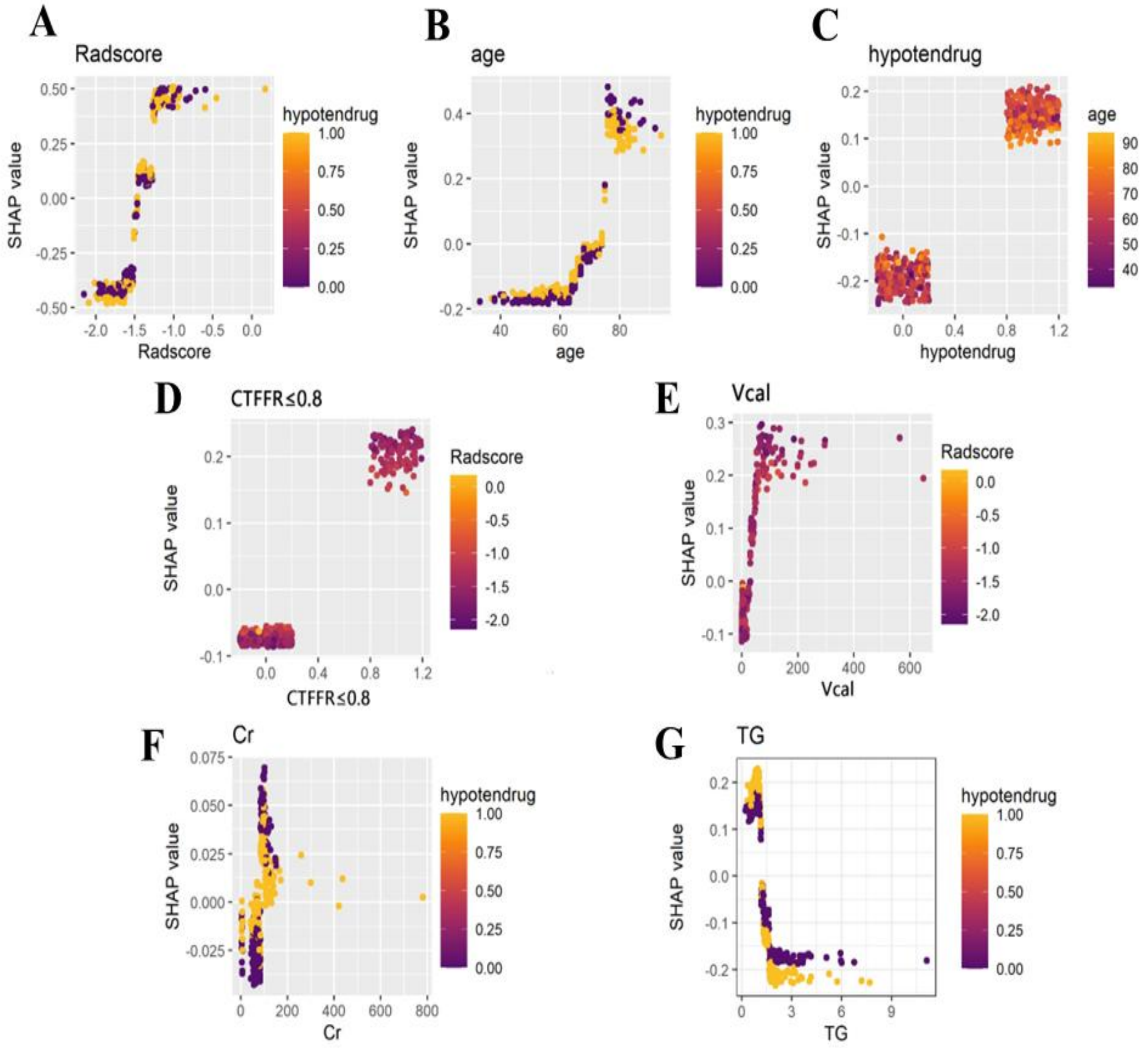

Supplemental Fig. C3 SHAP dependence plots for the non-diabetic group. SHAP, SHapley Additive exPlanations; Radscore, radiomic score; CTFFR, computed tomography-derived fractional flow reserve; CACS, coronary artery calcium score; NRS, napkin-ring sign; Cr, creatinine; TG, triglycerides; Vcal, calcified plaque volume.

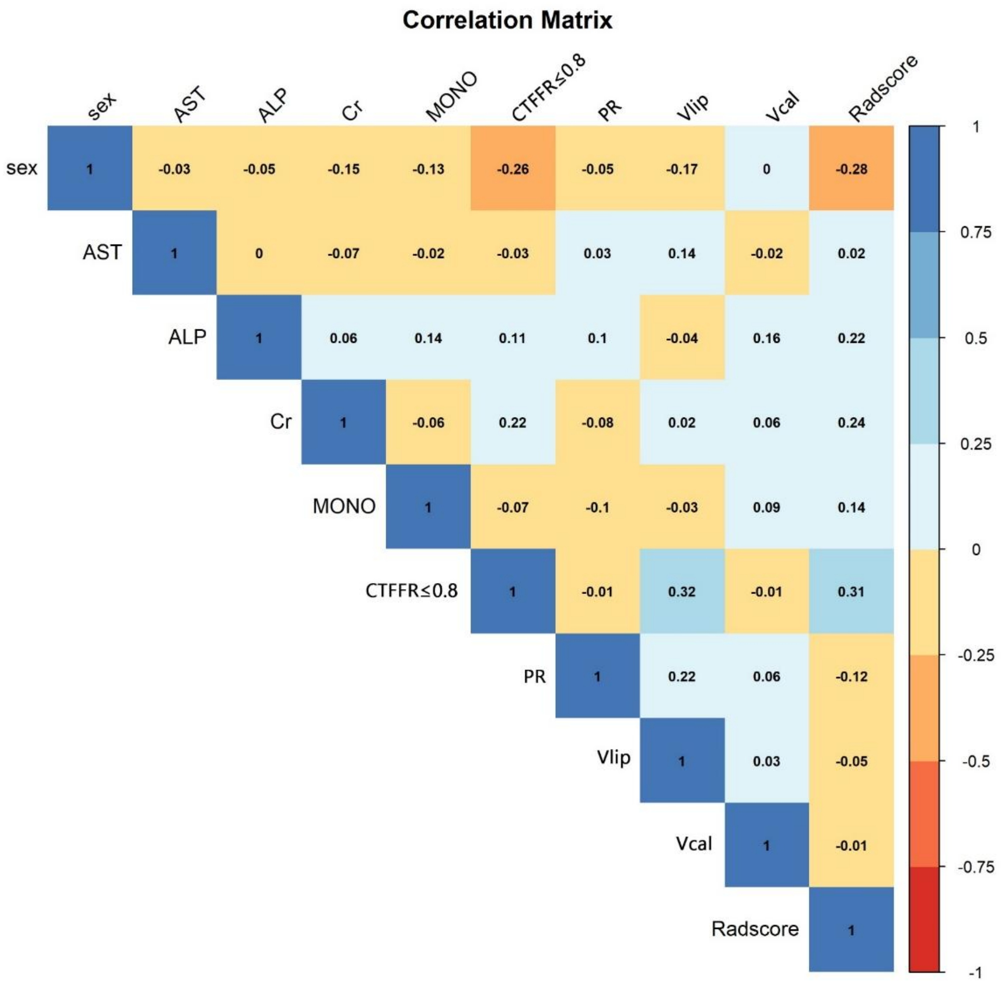

Supplemental Fig. C4 Correlation matrices for the diabetic group. Radscore, radiomic score; CTFFR, computed tomography-derived fractional flow reserve; PR, positive remodeling; AST, aspartate aminotransferase; ALP, alkaline phosphatase; Cr, creatinine; MONO, monocytes; Vlip, lipid plaque volume; Vcal, calcified plaque volume.

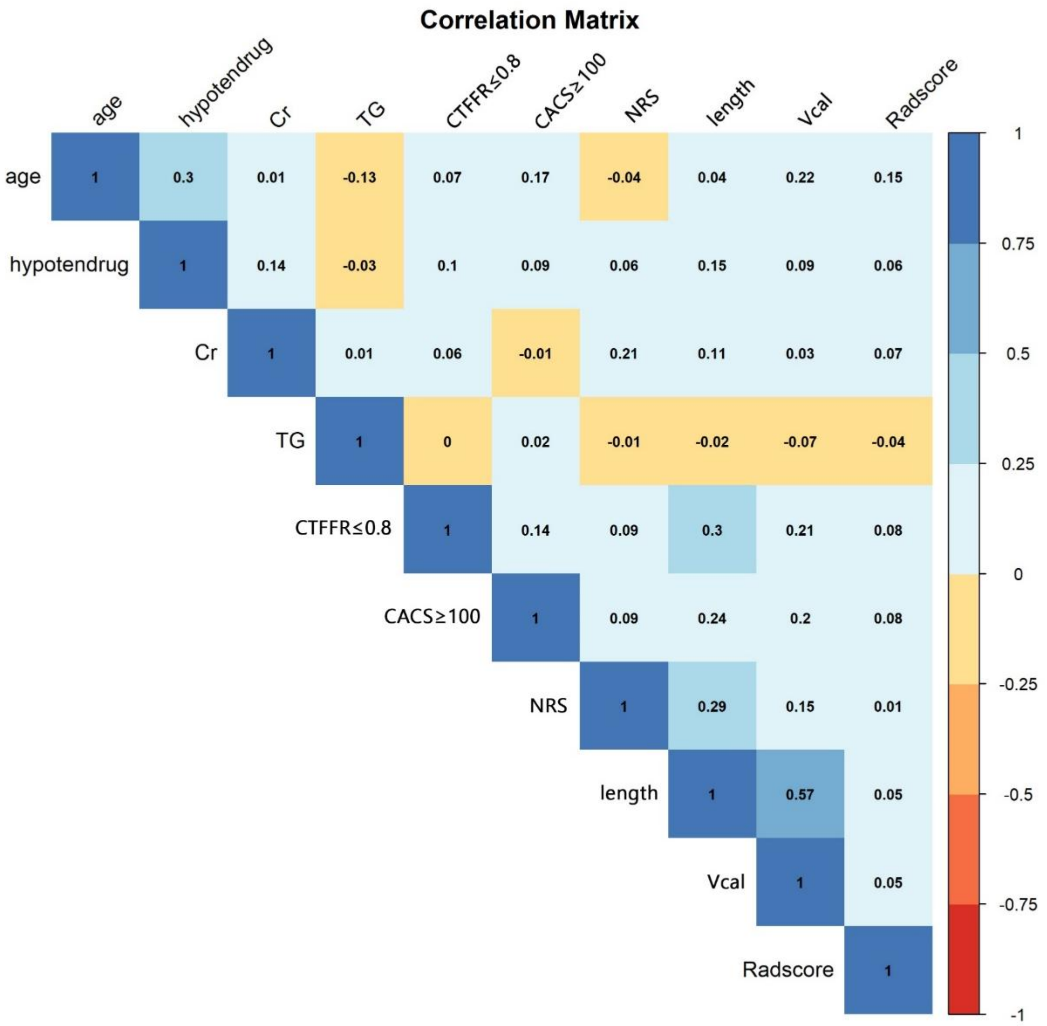

Supplemental Fig. C5 Correlation matrices for the non-diabetic group. Radscore, radiomic score; CTFFR, computed tomography-derived fractional flow reserve; CACS, coronary artery calcium score; NRS, napkin-ring sign; Cr, creatinine; TG, triglycerides; Vcal, calcified plaque volume.

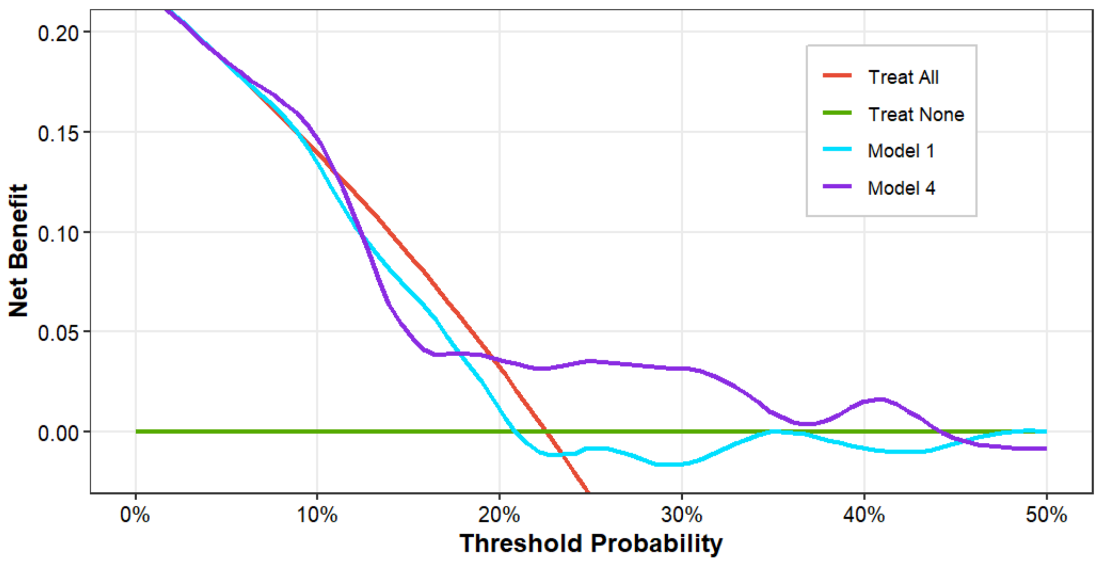

Supplemental Fig. C6 Decision Curve Analysis for 36-month survival prediction in the diabetic group. The y-axis measures the net benefit, calculated by summing the benefits (true positives) and subtracting the weighted harms (false positives). The x-axis represents the threshold probability for defining high risk. The red line represents the assumption that all patients have the event ("Treat All"), while the green line assumes no patients have the event ("Treat None").

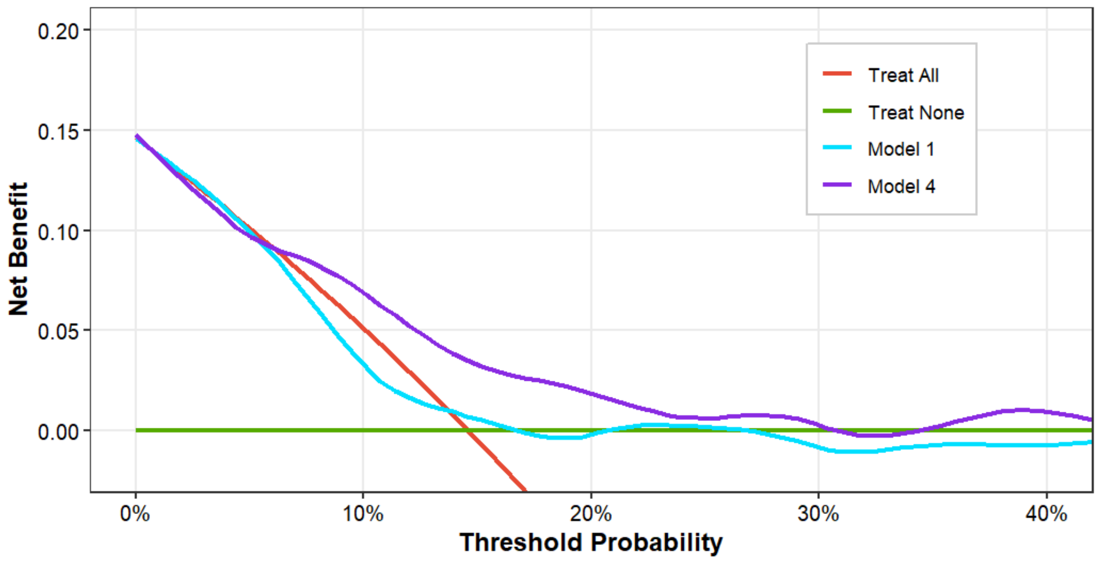

Supplemental Fig. C7 Decision Curve Analysis for 36-month survival prediction in the non-diabetic group. The y-axis measures the net benefit, calculated by summing the benefits (true positives) and subtracting the weighted harms (false positives). The x-axis represents the threshold probability for defining high risk. The red line represents

the assumption that all patients have the event ("Treat All"), while the green line assumes no patients have the event ("Treat None").

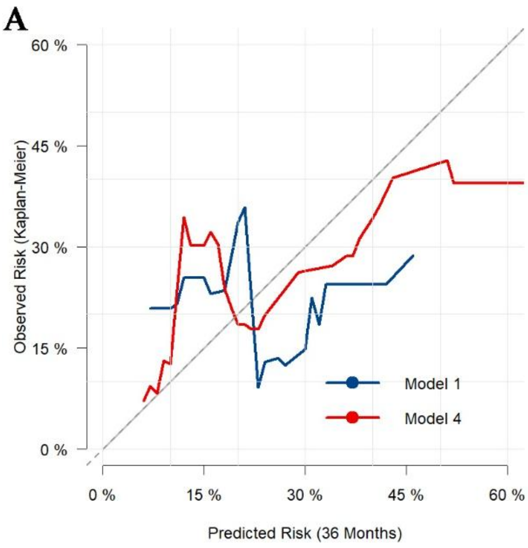

Supplemental Fig. C8 Calibration curves of the Clinical and Combined XGBoost models for 36 months in the diabetic group (A) and non-diabetic group (B). The x-axis represents the predicted 36-month mortality risk, and the y-axis represents the observed risk estimated by the Kaplan-Meier method. The dashed gray line indicates perfect calibration.

References

[1] 2. Diagnosis and Classification of Diabetes: Standards of Care in Diabetes-2024, Diabetes Care 47(Suppl 1) (2024) S20-s42.https://doi.org/10.2337/dc24-S002
